# Supplementary material for: Interaction between Risk Single-Nucleotide Polymorphisms of Developmental Dyslexia and Parental Education on Reading Ability: Evidence for Differential Susceptibility Theory
Source: Behav Sci (Basel). 2024 Jun 19;14(6):507. doi: 10.3390/bs14060507 (PMC11201191; doi:10.3390/bs14060507)
Supplement: Supplementary file 1 [file behavsci-14-00507-s001.zip › behavsci-2977637-supplementary.pdf]

Interaction between risk SNPs of Developmental Dyslexia and Parental Education on Reading Ability in Chinese Children: Evidence for Differential-Susceptibility Theory

Table S1. The selection of SNPs 2

Table S2. Correlation matrix of Cumulative Genetic Scores, SNPs,Character recognition, and PE. 3

Table S3. Results of the other 7 SNPs multiple regression on character recognition. 4

Table S4. Results for alternative regression models for the CGS of 8SNPs on reading ability. 5

Table S1. The selection of SNPs.

Lead SNP[a]	SNPs	LD(r^2)[b]	*P*	A1	Traits	T or I
**rs1541518**	rs2284225	0.8183	6.42E-08	G	Non-word reading	I
	rs4723046	0.8162				
	rs58094779	0.4553				
	rs4723047	0.827				
	rs741057	0.7959				
**rs281238**	rs1692778	0.8694	1.27E-07	T	Phoneme awareness	I
	rs281239	0.8711				
	rs281236	0.8702				
	rs281222	0.8645				
	rs281230	0.8670				
	rs281232	0.8702				
	rs281226	0.8678				
	rs281228	0.8688				
	rs281237	0.8711				
	rs281235	0.8688				
	rs166835	0.7876				
	rs281223	0.7773				
	rs4775684	0.7417				
	rs56167035	0.7411				
	rs12595418	0.7457				
	rs2059476	0.7411				
	rs1390873	0.7435				
	rs2132662	0.7427				
	rs59815685	0.7403				
**rs4571421**	rs4307051	0.9920	3.10E-07	C	Rapid automatized naming of pictures	I
**rs7301219**	NULL		5.43E-07	C	Rapid automatized naming of pictures	T
**rs9925265**	NULL		4.51E-07	G	Phoneme awareness	I
**rs764255**	rs8059199	0.9149	1.80E-07	T	Word reading	I
**rs6963842**	NULL		2.35E-07	G	Rapid automatized naming of letters	I
**rs9540938**	NULL		5.00E-07	A	Latent naming speed	I
**rs7187223**	NULL		9.90E-08	A	Non-word reading	I
**rs1555839**	rs7913742	0.7576	1.00E-09	Not present	RAS Letters/Numbers	
	rs10749593	0.9432				
	rs701825	0.585				
**rs17663182**	rs17605546	0.8455	4.77E-07	Not present	Rapid automatized naming of digit	
**rs349045**	NULL		8.00E-09	Not present	Reading and Spelling ability	
**rs16928927**	NULL		2.25E-08	Not present	Rapid automatized naming of letters	
**rs701825**	NULL		6.00E-08	Not present	RAS Letters/Numbers	
**rs2192161**	rs909257	0.993	7.34E-08	Not present	Non-word reading	
	rs2822560	0.5148				
	rs17002266	0.5853				
**rs113424746**	NULL		3.00E-07	Not present	RAS Letters/Numbers	
**rs6090818**	rs2021211	0.8551	3.14E-07	Not present	Word reading	
**rs76161559**	NULL		3.13E-07	Not present	Rapid automatized naming of digit	
**rs34525745**	NULL		3.20E-07	Not present	Non-word reading	
**rs4839516**	NULL		3.62E-07	Not present	Word reading	
**rs74702439**	rs77160407	0.6986	5.00E-07	Not present	Reading and Spelling ability	
**rs200580547**	NULL		4.76E-07	Not present	Rapid automatized naming of letters	

Note: a:according to LD, 22 lead SNPs are selected, and 9 SNPs present in the data.
b: LD: Linkage Disequilibrium, r^2 = 1 refers to completely linkage disequilibrium.
The red bold indicates that this SNP is present in the data.
T:genotyping; I: Imputed.

Table S2. Correlation matrix of Cumulative Genetic Scores, SNPs,Character recognition, and PE.

	1	2	3	4	5	6	7	8	9	10	11	12
1.Character recognition	1											
2.Parental education	.113***	1										
3.Cumulative Genetic Scores(CGS)	-.014	.001	1									
4.rs1541518	.004	.054*	.376***	1								
5.rs281238	.001	.000	.441***	.041	1							
6.rs4571421	-.014	.017	.413***	.042	.043	1						
7.rs7301219	-.038	.016	.366***	.024	-.023	.021	1					
8.rs9925265	-.006	-.027	.366***	-.006	.047	-.019	.029	1				
9.rs764255	-.017	0.15	.346***	.019	.029	.004	-.006	-.008	1			
10.rs6963842	.017	-.040	.263***	-.042	-.006	-.043	-.021	-.013	-.022	1		
11.rs9540938	.011	-.041	.187***	-.018	.058	.010	.021	-.010	.020	.005	1	
12.rs7187223	.019	-.027	.210***	-.002	-.016	-.015	.009	.017	.006	-.003	-.015	1

Note: [a]Information collected from original studies. [b]Results analyzed from current study.

Table S3. Results of the other 7 SNPs multiple regression on character recognition.

Standard parameterization: Main effects and G×E interaction																
Parameter	B_0	*p*	B_1	*p*	B_2	*p*	B_3	*p*	B_4	*p*	B_5	*p*	R^2	*F*	*df*	*p*
rs1687482	-8.42(6.37)	.19	2.18(.94)	.02	.58(2.06)	.78	-.22(.59)	.71	.92(.04)	<.001	-2.55(.95)	.007	.2527	100.8	1471	<.001
rs6963842	-7.00(5.80)	.23	1.61(.52)	.002	-1.44(2.40)	.55	.66(.70)	.35	.92(.04)	<.001	-2.58(.95)	.007	.2534	101.2	1471	<.001
rs8188533	-11.10(7.90)	.16	3.09(1.67)	.06	1.93(3.12)	.54	-.68(.91)	.45	.92(.04)	<.001	-2.56(.95)	.007	.2529	100.9	1471	<.001
rs9540938	-11.88(10.26)	.25	2.60(2.41)	.28	2.16(4.46)	.63	-.38(1.25)	.76	.92(.04)	<.001	-2.59(.95)	.006	.2528	100.9	1471	<.001
rs7187223	-7.75(5.70)	.17	1.88(.46)	<.001	.85(3.52)	.81	.06(1.04)	.95	.92(.04)	<.001	-2.55(.95)	.007	.2530	101.0	1471	<.001
rs10010152	-12.23(6.92)	.08	3.18(1.27)	.01	2.86(2.46)	.24	-.81(.73)	.27	.92(.04)	<.001	-2.54(.95)	.007	.2533	101.1	1471	<.001
rs764255	-8.36(5.75)	.15	2.26(.52)	<.001	2.23(2.31)	.33	-.82(.64)	.20	.92(.04)	<.001	-2.56(.95)	.007	.2537	101.4	1471	<.001

Table S4. Results for alternative regression models for the CGS of 8SNPs on reading ability.

Standard parameterization			Re-parameterized regression equation		
Parameter	Gene(G) and environment(E) main effects: Model 5	Main effects and G×E interaction: Model 6	Paramete	Differential susceptibility Model 3g	Diathesis-Stress Model 3h
A_0	-6.58(5.96)	2.67(7.47)	C	3.60(.62)	6.84(--)[a]
A_1	1.87(.43)	-.93(1.43)	A_0	-0.68(5.07)	5.37(4.59)
A_2	-.16(.28)	-1.69(.80)	A_1	-0.93(1.43)	1.34(.62)
A_3	-	.47(.23)	A_2	.47(.23)	.09(.07)
A_4	.92(.04)	.92(.04)	A_3	0.92(.04)	.92(.04)
A_5	-2.57(.95)	-2.58(.95)	A_4	-2.58(.95)	-2.58(0.95)
R^2	.2553	.2549	R^2	0.2554	0.2538
F	126.20	102	F	127.60	126.50
df	4,1472	5,1471	df	4,1472	4,1472
p	<.0001	<.0001	p	<.0001	<.0001
F vs. 5	--	4.21	F vs. 3h	3.09	--
df	--	1472	df	1472	--
p	--	0.0403	p	.0788	--
AIC	12770.24	12768.02	AIC	12768.02	12769.13
BIC	12802.03	12805.11	BIC	12805.11	12800.9

Note: AIC, Akaike information criterion; BIC, Bayesian information criterion.

Tabled values are parameter estimates, with their standard errors in parentheses.

F vs. 5, 3h stands for an *F* test of the difference in R^2 for Model 6 versus Model 5, Model 3g versus Model 3h, respectively.

[a] Parameter fixed at reported value; SE is not applicable, so is listed as (--).
